# Supplementary figures and images for: Genome-Wide Association Study for Screening and Identifying Potential Shin Color Loci in Ducks
Source: Genes (Basel). 2022 Aug 4;13(8):1391. doi: 10.3390/genes13081391 (PMC9407491; doi:10.3390/genes13081391)

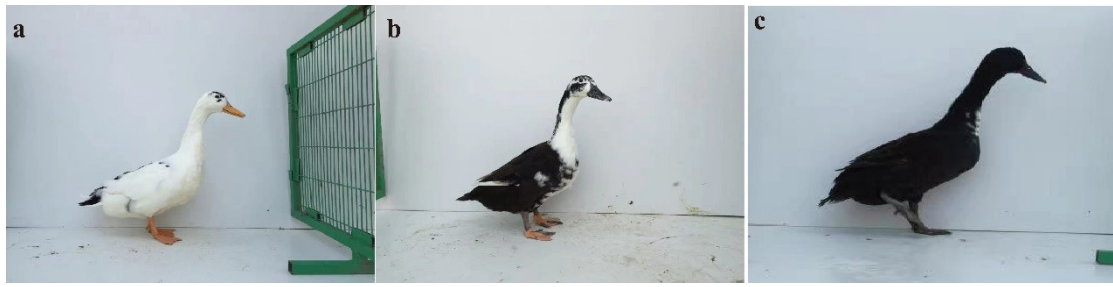

**Figure S1.** Different shin color duck.

Supplement: Supplementary file 1 [file genes-13-01391-s001.zip › Figure S1 Duck different shin color.pdf]
